# Histology: Normal and Morbid

**Published:** 1899-04

**Authors:** 


Histology: Normal and Morbid. By Edward K. Dunham, Ph. B., M. D., Professor of General Histology, Bacteriology and Hygiene in the University and Bellevue Hospital Medical College, New York. Illustrated by 363 engravings. Published by Lea Bros. & Co., New York and Philadelphia. Pages, 448. Price, cloth, $3.25, net.

In the preface of the work before us, the author makes an apology for attempting the discussion of so large a subject in such small space, but his apology is entirely unnecessary. He should remember that none except the younger set of the present generation of general practitioners had access during their college days to superbly equipped physiological and pathological laboratories, or lectures and demonstrations by a corps of trained and experienced microscopists, as the students of the present time have. Indeed, the better half of us have no knowledge upon these subjects save what we have gleaned from discussions of special subjects in text-books and journals. Hence, our knowledge is fragmentary and unsatisfactory. It cannot be expected that a busy practitioner will take the time to read any extensive work, full of technicalities and experimental studies, upon a subject which, though vastly important as it is, does not bear directly upon his daily work. Therefore the work before us is timely and valuable. It contains the cream of the subject with all non-essentials left out. It is written in terse, didactic style, and will be of great benefit to every general practitioner and student who will read it. It will prove of especial benefit to those who have a microscope and are not thoroughly trained in its use. J.

